# Large scale analysis of pediatric antiviral CD8+ T cell populations reveals sustained, functional and mature responses

**DOI:** 10.1186/1742-4933-3-11

**Published:** 2006-12-08

**Authors:** Haruki Komatsu, Ayano Inui, Tsuyoshi Sogo, Tomoo Fujisawa, Hironori Nagasaka, Shigeaki Nonoyama, Sophie Sierro, John Northfield, Michaela Lucas, Anita Vargas, Paul Klenerman

**Affiliations:** 1 Department of Pediatrics, National Defense Medical College, Saitama, Japan; 2 Department of Pediatrics, Toho University Sakura Hospital, Chiba, Japan; 3 Department of Pediatrics, Atami Hospital, International University Health and Welfare, Shizuoka, Japan; 4 Department of Pediatrics, Hokkaido University Graduate School of Medicine, Hokkaido, Japan; 5 Nuffield Department of Medicine, Peter Medawar Building for Pathogen Research, University of Oxford, Oxford, UK

## Abstract

**Background:**

Cellular immunity plays a crucial role in cytomegalovirus (CMV) infection and substantial populations of CMV-specific T cells accumulate throughout life. However, although CMV infection occurs during childhood, relatively little is know about the typical quantity and quality of T cell responses in pediatric populations.

**Methods:**

One thousand and thirty-six people (Male/Female = 594/442, Age: 0–19 yr.; 959 subjects, 20–29 yr.; 77 subjects) were examined for HLA typing. All of 1036 subjects were tested for HLA-A2 antigen. Of 1036 subjects, 887 were also tested for HLA-A23, 24 antigens. In addition, 50 elderly people (Male/Female = 11/39, Age: 60–92 yr.) were also tested for HLA-A2 antigen. We analyzed the CD8+ T cell responses to CMV, comparing these to responses in children and young. The frequencies, phenotype and function CD8+ T cells for two imunodominant epitopes from pp65 were measured.

**Results:**

We observed consistently high frequency and phenotypically "mature" (CD27 low, CD28 low, CD45RA+) CMV-specific CD8+ T cell responses in children, including those studied in the first year of life. These CD8+ T cells retained functionality across all age groups, and showed evidence of memory "inflation" only in later adult life.

**Conclusion:**

CMV consistently elicits a very strong CD8+ T cell response in infants and large pools of CMV specific CD8+ T cells are maintained throughout childhood. The presence of CMV may considerably mould the CD8+ T cell compartment over time, but the relative frequencies of CMV-specific cells do not show the evidence of a population-level increase during childhood and adulthood. This contrast with the marked expansion ("inflation") of such CD8+ T cells in older adults. This study indicates that large scale analysis of peptide specific T cell responses in infants is readily possible. The robust nature of the responses observed suggests vaccine strategies aimed at priming and boosting CD8+ T cells against major pathogens (including HIV, malaria and CMV itself) could be successful in this age-group.

## Background

Human cytomegalovirus (HCMV) is a widespread β-herpesvirus that infects 40% to 100% of adults worldwide [1]. The common transmission mode of HCMV is vertical: in almost all cases, HCMV infection occurs during prenatal period (transplacental acquisition), at birth (through exposure to HCMV in the birth canal), or postnatal period (including transmission by breast milk) [2,3]. In developed countries, seroconversion for HCMV occurs throughout life. Primary infection is largely asymptomatic and HCMV infection persists lifelong usually without clinical sequelae in healthy individuals. However, recrudescence of latent infection can cause life-threatening diseases in immunocompromised host, such as transplant recipients, patients receiving tumor therapy, and patients with advanced human immunodeficiency virus infection [1,4]. Moreover, recent studies have shown that primary HCMV infection can produce severe disease in premature infants [3,5]. Therefore, studies of human immune responses to HCMV are required to understand the mechanisms involved in control of infection and develop improved strategies to prevent CMV-induced disease.

Viral infection is usually controlled by a range of innate and adaptive immune effector mechanisms. In particular, virus-specific CD8+ cells play a crucial role in the control of HCMV replication during both acute and chronic phases [6-8]. Strong virus-specific CD8+ T cell responses develop during primary infection and functional HCMV-specific CD8+ T cells are present after resolution of primary infection. Thereafter, these HCMV-specific CD8+ T cells are maintained at high frequencies. The proportion of the HCMV-specific CD8+ T cells in the total lymphocyte pool may reach very high levels in elderly individuals [9]. In murine models, accumulation of murine cytomegalovirus (MCMV) -specific CD8+ T cells was seen in all organs, starting some time after resolution of primary infection [8]. This phenomenon, termed "memory inflation", was also observed in human adults [9]. These observations are consistent with the idea that continuous or repetitive exposure to antigens slowly moulds memory T cell populations over time.

The "phenotype" of virus-specific CD8+ T cells can be described using a few distinct subsets based on cell surface markers (CD28, CD27, CD45RA, and CD45RO). CD27 and CD28 are T cell costimulatory molecules, which are differentially expressed on CD8+ T cells under different conditions of stimulation over time [10-13]. Naïve and so-called "early-differentiated" cells are characterized by high expression of the costimulatory molecules CD28 and CD27, which may progress through CD28-CD27+ and on to CD28-CD27- cells, typical of both HCMV and MCMV, considered to be at a "late" stage of differentiation [14]. Similarly, isoforms of CD45, leukocyte common antigen, can be used as a cell surface marker to examine differentiation status. In human cord blood, almost all CD8+ cells show high expression of CD45RA, while the proportion of cells expressing CD45RO increases with age [15]. HCMV-specific CD8+ cells show high expression of CD45RO during primary HCMV infection, but are relatively enriched in CD45RA+ cells during the chronic phase of HCMV infection [14,16,17]. This is thought to correlate either with a "terminally differentiated" status, or entry into a program of a long term quiescence and survival.

Little detail is available on the frequencies, phenotype and function of antiviral CD8+ T cells in pediatric populations. It was previously considered that infants may fail to mount Th1 type responses of a quality similar to those seen in adults, although emerging data from smaller studies has led to a revision of these ideas. A recent study showed that a mature and functional CD8+ T cell response to HCMV was observed in specific cases of infection of fetuses and newborns [18]. However, it is unclear what the normal response is in larger, asymptomatically infected pediatric populations, and how these populations evolve through childhood. Immaturity of CD4+ T cell help, regulation, dendritic cells or innate immunity could all potentially have an important impact on long-term maintenance of antiviral CD8+ T cell populations. Furthermore, several features of the expanded populations of CD8+ T cells associated with persistent virus infection have been likened to "ageing", and thus study of such CD8+ T cells in an infant population might provide some insights into the induction and maintenance of "normal" memory in adults [19]. Analysis of pediatric populations has been hindered to date by largely by limitations on the blood sampling available, but recent advances in analysis of CD8+ T cells has meant high quality data may be obtained from as little as 100 μl of blood [9].

In this study, a large cross-sectional analysis was performed to clarify the status of HCMV specific CD8+ T cells in infants and children. In order to quantify HLA-restricted HCMV-specific CD8+ T cells, we used human leukocytic antigen (HLA)-A2 and HLA-A24 tetrameric complexes (tetramers) loaded with the immunodominant peptide derived from HCMV structural protein pp65. In addition, effector functions of HCMV-specific CD8+ T cells in children were assessed using IFN-γ enzyme-linked immunospot (ELISPOT) assay and intracellular cytokine staining (ICS) for IFN-γ-producing cells. Furthermore, we evaluated the differentiation status using cell surface markers (CD28, CD27, CD45RA, and CD45RO) to clarify the phenotype of the HCMV-specific CD8+ T cells and any changes over time. We show that the effector functions of HCMV-specific CD8+ cell in infants are as strong as those in young adults. Moreover, the phenotype of HCMV-positive CD8+ T cells is well differentiated in infants and young children.

## Materials and methods

### Study population

Between March and September 2003, blood samples were taken from patients who attended pediatric clinics at National Defense Medical College (Tokorozawa, Japan), International University Health and Welfare (Atami, Japan), and Hokkaido University (Sapporo, Japan) due to various diseases. Informed consent was taken from them or their parents. One thousand and thirty-six people (Male/Female = 594/442, Age: <1yr.; 132, 1–5yr,; 305, 6–10yr.; 216, 11–15yr.; 218,; 16–19 yr.; 88, 20–29 yr.; 77) were enrolled in this study. All of 1036 subjects were tested for HLA-A2 antigen. Of 1036 subjects, 887 (Male/Female = 519/368, Age: <1yr.; 115, 1–5yr,; 279, 6–10yr.; 190, 11–15yr.; 172,; 16–19 yr.; 68, 20–29 yr.; 63) were also tested for HLA-A23, 24 antigens. In addition, 50 elderly volunteers (Male/Female = 11/39, 60–69yr.; 6, 70–79yr.; 8, 80–89yr.; 22, 90–92 yr.; 14) were also enrolled in this study. None of them was examined for HCMV sero -status.

### Monoclonal Abs and peptides

Anti-HLA-A2-fluorescein isothiocyanate (FITC), anti-CD8-peridinin chlorophyll protein (PerCP), anti-CD27-FITC, anti-CD28-:allophycocyanin (APC), anti-CD45RA-FITC, anti-CD45RO-APC, anti-IFN-γ-FITC mAbs, and isotype control Abs were purchased from Becton Dickinson PharMingen (San Diego, CA, USA). Anti-HLA-A23, 24 mAbs were purchased from One Lambda (Canogo Park, CA, USA). Goat anti-mouse IgG-FITC Abs were purchased from Immunotech (Marseille, France). The HLA-A*0201-restricted HCMV-specific CTL epitope pp65 495–503 (NLVPMVATV) and HLA-A*2402 restricted HCMV-specific CTL epitope pp65 328–336 (QYDPVAALF) both previously identified [20,21], were purchased from MBL (Nagoya, Japan).

### HLA-peptide tetrametric complexes

HLA class I-peptide tetramers were synthesized as described previously [22]. Briefly, recombinant HLA class I proteins (HLA-A*0201 and HLA-A*2402) were expressed and produced in Escherichia coli. The A24-expressing plasmid was provided by Dr. Kuzushima (Aichi Cancer Center Research Institute, Japan) refolded with β2-microglobulin and peptide. After refolding of the HLA class I molecle, β2-microglobulin and peptide, the complex was purified by FPLC and biotinylated by Bir A enzyme (Avidity, Denver, CO, USA). HLA class I-peptide complexes were mixed with phycoerythrin (PE)-labeled streptavidin at a molar ratio of 4: 1 to form HLA-peptide tetrametric complexes. Tetramers were titrated against peripheral mononuclear cells (PBMCs) from HCMV-seropositive and mismatched donors to determine the concentration that induced maximal staining with minimal background.

### Cell surface and intracellular staining

#### HLA typing

Detection of HLA-A2 and HLA-A24 was performed by flow cytometry using the HLA-A2 and HLA-A23, 24 mAb. In brief, 20 μL of EDTA or heparinized whole blood was stained with anti-HLA-A2-FITC and HLA-A23, 24 mAb for 20 minutes at 4°C respectively. After staining with anti-HLA-A2-FITC mAb, red blood cells were lysed using FACS lysis (Becton Dickinson Sciences, San Jose, CA, USA). Whole blood stained with anti-HLA-A23, 24 mAb was washed with PBS. After the incubation for 20 minutes at 4°C with FITC-labeled mouse IgG, it was washed again with PBS and lysed using FACS lysis solution (Becton Dickinson Sciences).

#### Tetramer staining

50 μL of whole blood was stained with tetramer for 15 minutes at 37°C, followed by addition of titrated Abs (FITC-, PerCP-, or APC- conjugated) directed against surface molecules and incubation for 20 minutes at 4°C. Samples were lysed as described above.

#### Intracellular cytokine staining

To determine the frequencies of IFN-γ-producing peptide-specific T cells in peripheral blood, intracellular staining was conducted on fresh whole blood. Frequencies of IFN-γ-producing peptide-specific T cells were quantified after stimulation with the CMV HLA-A2 peptide (pp65 495–503, NLVPMVATV), according to the protocol of the supplier (Becton Dickinson Bioscience, San Jose, CA, USA). Briefly, heparinized whole blood were stimulated with peptide (at 10 μM final concentration) for 6h at 37°C in the presence of Brefeldin (Sigma-Aldrich) at 10 μL/ml. Whole blood was stained with anti-CD8-PerCP Ab for 20 minutes at room temperature. Whole blood was lysed and fixed using FACS lysis solution (Becton Dickinson PharMingen), permeabilized using FACS permeabilization buffer (Beckton Dickinson), and then stained with FITC-labeled IFN-γ or FITC labeled IgG-isotype control mAb.

Cell were stored in Cell Fix buffer (Becton Dickinson Immunocytometry systems) at 4°C until analysis. Samples were analyzed with a FACS Calibur (Becton Dickinson Bioscience) using CellQuest software (Becton Dickinson Bioscience), after compensation was checked using freshly stained PBMCs.

### Elispot assays

PBMCs were isolated from heparinized venous blood by density gradient sedimentation using Ficoll-Hypaque (Lymphoprep; Axis Shield, Oslo Norway). Fresh PBMCs were plated in 96-well polyvinylidene plates (Milipore, Bedford, MA, USA) that have been precoated with 0.5 μg/ml anti-IFN-γ-mAb (Mabtech, Stockholm, Sweden). The peptides were added in a volume of 10 μl and then PBMCs were added at 50,000–100,000 cells/well in a volume of 190 μl. The final concentration of HLA-A2 peptide (NLVPMVATV) was 5 μM. The plates were incubated overnight at 37°C, 5% CO2 and washed PBS before addition of the second, biotinylated anti-IFN-γ mAb (Mabtech) at 0.5 μg/ml and incubated at room temperature for 100 min. After washing, streptavidin-conjugated alkaline phosphatase (Mabtech) was added at room temperature for 40 minutes. Individual cytokine-producing cells were detected as dark spots after 20-minutes reaction with 5-bromo-4-chloro-3-indolyl phosphate and nitro blue tetrazolium using an alkaline phosphatase-conjugated substrate (Bio-Rad Laboratories, Hercules, CA, USA). The number of specific T cells was calculated by subtracting the negative control values and expressed as either the number of spot-forming units per 10^6 ^PBMC.

### Statistical analysis

Frequency distributions were compared using the Yates corrected chi-square test or Fisher's exact test. A *p *value of 0.05 or less was considered to indicate statistical significance. For nonparametric statistical analyses, Spearman rank correlation test were used. All *p *values were 2-tailed and considered significant if <0.05. Nonparametric Mann-Whitney *U *tests were used for data pairs and Kruskal-Wallis tests were used for group data. Results were presented using Prism (GraphPad, San Diego, CA, USA).

## Results

### Frequency of HLA-A2 and A24

Of 1036 subjects, 448 (43%) were positive for HLA-A2. Of 887 subjects, 519 (58%) people were positive for HLA-A23, 24, of which 76 (15%) were also positive for HLA-A2. Because it is well established that HLA-A23 antigen is very rarely detected in Japanese populations [23,24], the HLA-A23, 24-positive subjects were considered to be positive for HLA-A24. To compare with children and young adults, additionally 50 elderly people were enrolled in this study. All of them were in good health. Of these elderly people, 21 (42%) were positive for HLA-A2.

### Frequency of HCMV tetramer-positive responses at different ages

**Figure 1:**
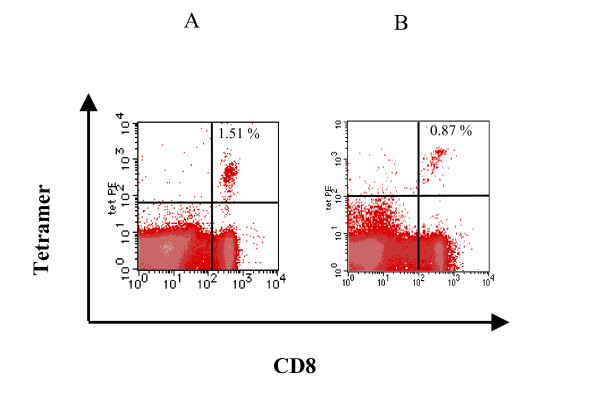
**Example of CMV tetramer staining in infants**. A symptomatic 2-month-old HLA-A2+ infant (A) and an asymptomatic 1-month-old HLA-A24+ infant (B) were stained with pp65 tetramer. The plot shown is gated on live lymphocytes and reveals a frequency of HCMV-specific CD8+ T cells. HLA-A2 tetramer/CD 8+ lymphocytes was 1.51% in the HLA-A2 infant and 0.87% in the HLA-A24+ infant.

Initially, a cross-sectional analysis of HCMV-specific CD8+ T cell frequency was performed. Analysis of HLA-A2 negative and HLA-A23, 24 negative samples provided a cut-off for detection of 0.05% and 0.04% of CD8, respectively, as previously obtained [9] (data not shown). Fig 1 shows an example of positive tetramer staining in HLA-A2+ and HLA-A24+ infants. Of 448 HLA-A2-positive subjects, HCMV-A2 tetramer staining revealed positive responses in 190 (42%). Of 519 HLA-A23, 24-positive subjects, HCMV-A24 tetramer staining revealed positive responses in 133 (26%).

Table 1 shows the results of the HCMV-A2 tetramer assay in individual age-groups. The percentage of the HLA-A2 subjects, who were positive for HCMV-A2 tetramer, was 31% in 0–4 yr. group, and increased gradually with age, such that approximately half the adults and elderly populations were positive. However, the mean percentage of CD8+ T cells stained with HCMV-A2 tetramer did not show a continuous increase with age. Indeed, the mean percentage of CD8+ T cells stained with HCMV-A2 tetramer of the 0–4 yr. group was slightly higher than that of the 5–9 yr. group and the 10–14yr. group. In order to confirm that elderly individuals could show a high frequency of HCMV specific CD8+ T cells in Japan, 50 elderly individuals were examined. The mean frequency of HCMV-specific CD8+ T cells in elderly group was as high as that in the previous study [9] and significantly higher than that in any other groups.

**Table 1:** Summary of HCMV-tetramer in HLA-A2 individual groups

Age(yr.) (n = 469)	0–4 (n = 179)	5–9 (n = 97)	10–14 (n = 94)	15–19 (n = 50)	20–29 (n = 28)	60–92 (n = 21)
Number of tet(+)	55(31%)	43(44%)	49(52%)	28(46%)	15(54%)	12(57%)
CD8(%)	18.9 ± 4.7	21.5 ± 7.4	20.2 ± 6.9	20.3 ± 6.7	24.8 ± 7.1	23.2 ± 9.6
Tet(%)	0.19 ± 0.23	0.18 ± 0.25	0.22 ± 0.31	0.23 ± 0.19	0.41 ± 0.54	1.49 ± 1.79
Tet/CD8(%)	1.05 ± 1.30	0.85 ± 1.04	1.02 ± 1.36	1.14 ± 0.96	1.55 ± 1.71	5.76 ± 4.19

HCMV-tetramer positive: Tet/CD8(+) Lymphocytes ≥ 0.05%

The results of HCMV-A24 tetramer staining are shown in Table 2. Although the percentage of HCMV-A24 tetramer positive subjects increased gradually with age, the percentage of the HLA-A24 tetramer positive subjects was 19% in the 0–4 yr. group, which was lower than that of comparable HLA-A2 tetramer positive subjects (31%), and this remained similar across all age groups. Unlike the HLA-A2-specific responses, an increase was not observed in the mean percentage of HLA-A24 tetramer-positive cells in young adults, although in this case an elderly population was not tested. Interestingly, as for the HLA-A2+ individuals, the mean percentage of CD8+ T cells stained with HCMV-A24 tetramer in the youngest group (0–4 yr.) was higher than that at later ages. Taken together, those findings suggest that young children have cellular immune responses to CMV comparable to those in young adults.

**Table 2:** Summary of HCMV-tetramer in HLA-A24 individual groups

Age(yr.) (n = 519)	0–4 (n = 202)	5–9 (n = 106)	10–14 (n = 108)	15–19 (n = 58)	20–29 (n = 45)
Number of tet(+)	39(19%)	32(30%)	23(21%)	24(41%)	15(33%)
CD8(%)	20.0 ± 5.7	18.6 ± 5.1	19.5 ± 6.9	22.3 ± 8.3	24.8 ± 6.4
Tet(%)	0.05 ± 0.07	0.02 ± 0.03	0.02 ± 0.01	0.04 ± 0.06	0.03 ± 0.03
Tet/CD8(%)	0.21 ± 0.31	0.12 ± 0.10	0.15 ± 0.24	0.14 ± 0.16	0.12 ± 0.10

HCMV-tetramer positive: Tet/CD8(+) Lymphocytes ≥ 0.04%

Seventy-six individuals were positive for both HLA-A2 and HLA-A24. Of 76 subjects, 17 were positive for both HCMV-A2 and -A24 tetramer staining. The mean frequency of HCMV-specific CD8+ T cells in HLA-A2 and HLA-A24 individuals was 1.09 and 0.09, respectively. Of 76 subjects, 12 and 8 were positive for HCMV-A2 and -A24 tetramer staining, respectively. The remainings were negative for trtramer staining. There was no significant difference in positive rate of HCMV tetramer between two alleles and one allele (HLA-A2, A24 vs. HLA-A2; *p *= 0.79, HLA-A2, A24 vs HLA-24; *p *= 0.15). These findings suggest that HLA type did not influence the immunodominance using tetramer assay.

### Analysis of CD8+ T cell responses in infants and young children

To assess in detail the results of HCMV tetramer staining in the key population of infants and young children, the youngest group was further divided by age and analyzed (Table 3, 4). Here, the percentage of HCMV tetramer positive subjects increased slightly with age. Under 1 year of age, 23% and 17% were positive for HCMV tetramer staining in HLA-A2 subjects and HLA-A24 subjects, respectively. The youngest subject who was positive for HCMV tetramer staining in the HLA-A2 and HLA-A24-positive population, were 2- and 1-months-old, respectively (Figure 1). Although the 2 month-old girl was referred to our hospital due to jaundice, the 1-month old baby had no symptoms. On the other hand, the mean percentage of HCMV tetramer-positive cells amongst total lymphocytes or amongst CD8+ T cells had a slight decrease with age, lowest at around 3–4 years. These findings are consistent with prevalent primary HCMV infection in early life was and confirm that strong HCMV-specific CD8+ T cell responses can be reproducibly generated in the first year of life.

**Table 3:** Summary of HCMV-tetramer in HLA-A2 young children

Age(yr.) (n = 179)	< 1 (n = 60)	1 (n = 45)	2 (n = 30)	3 (n = 27)	4 (n = 17)
Number of tet(+)	14(23%)	17(38%)	9(30%)	11(41%)	4(24%)
CD8	18.1 ± 5.1	16.7 ± 4.1	22.5 ± 3.5	19.7 ± 4.4	20.1 ± 5.1
Tet(%)	0.20 ± 0.20	0.25 ± 0.32	0.17 ± 0.19	0.15 ± 0.14	0.08 ± 0.05

Tet/CD8(%)	1.16 ± 1.13	1.48 ± 1.78*	0.73 ± 0.77*	0.75 ± 0.65*	0.28 ± 0.26*

HCMV-tetramer positive: Tet/CD8(+) Lymphocytes ≥ 0.05%

*: *P *values < 0.05

**Table 4:** Summary of HCMV-tetramer in HLA-A24 young children

Age(yr.) (n = 202)	< 1 (n = 66)	1 (n = 46)	2 (n = 38)	3 (n = 25)	4 (n = 27)
Number of tet(+)	11(17%)	11(24%)	7(18%)	4(16%)	6(22%)
CD8	17.0 ± 5.0	20.0 ± 6.9	19.2 ± 4.2	22.3 ± 3.8	24.8 ± 5.6
Tet(%)	0.07 ± 0.08	0.05 ± 0.05	0.06 ± 0.10	0.01 ± 0	0.03 ± 0.03

Tet/CD8(%)	0.34 ± 0.32*	0.16 ± 0.15*	0.28 ± 0.55*	0.05 ± 0.01*	0.11 ± 0.12

HCMV-tetramer positive: Tet/CD8(+) Lymphocytes ≥ 0.04%

*: *P *values < 0.05

### Correlation between tetramer and functional assays

**Figure 2:**
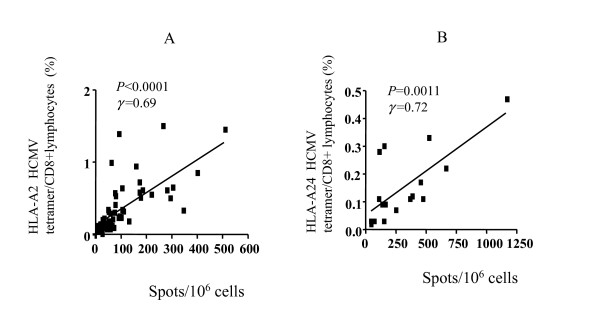
**Correlation of HLA-A2 HCMV tetramer staining and IFN-γ Elispot assay**. (A) 56 HLA-A2+ subjects were studied, including children and adults (age: from 3 months to 29 years old, mean ± SD; 10.5 ± 7.3 years). (B) 17 HLA-A24 subjects were studied including children and adults (age: from 1 month to 23 years old, mean ± SD; 8.9 ± 8.8 years).

**Figure 3:**
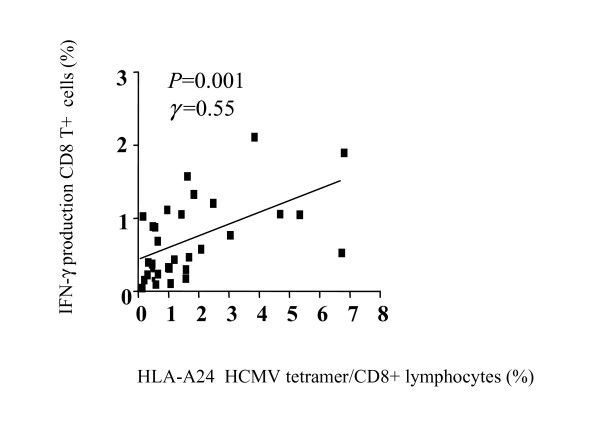
Correlation between intracellular interferon-γ staining and HLA-A2 HCMV tetramer positive cells/CD8+ lymphocytes. There were 33 subjects including children, and adults (age: from 2 months to 29 years old, mean ± SD; 10.1 ± 8.5 years).

**Figure 4:**
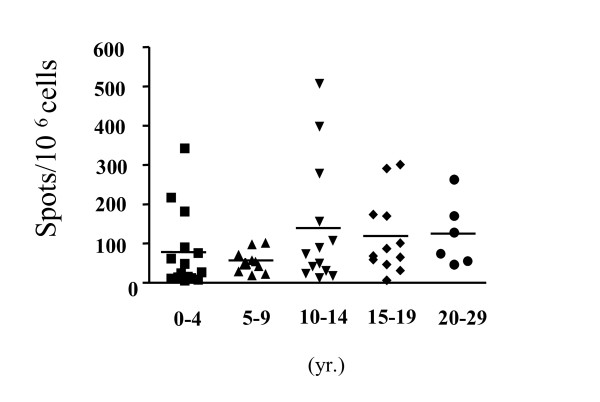
**IFN-γ ELISPOT assay of HLA-A2 individuals by age-group**. Individuals from different age groups as indicated were analyzed for HLA-A2 peptide specific responses using ELISPOT as previously. The differences shown were not significant.

To evaluate the effector function of the HCMV-specific cells, we performed IFN-γ ELISPOT assays and compared these with tetramer staining. Fifty-six HLA-A2+ subjects (age: from 3 months to 29 year-old, mean 10.5 years old) and 17 HLA-A24+ subjects (age: from 1 months to 23 years old, mean 8.9 years old) were selected for ELISPOT assays. A comparison of the ELISPOT assay and tetramer assay is shown in Figure 2. Although the number of HLA-A24+ subjects was small, the comparison of ELISPOT assay and tetramer assay demonstrates a strong association (HLA-A2: *p *< 0.0001, γ = 0.69 ; HLA-A24: *p *= 0.0011, γ = 0.72). We also performed ICS for IFN-γ for HLA-A2+ subjects (n = 33, age: from 2 months to 29 years old, mean 10.1 year-old). A comparison of the ICS and tetramer assays is shown in Figure 3. Although the association was not quite as strong as the correlation between ELISPOT assay and tetramer assay, a significant association was observed (γ = 0. 55, *p *= 0.001). The results of ELISPOT assays in HLA-A2 subjects were stratified by age and shown in Figure 4. The mean spot number of the 0–4 yr. group was 79.7/10^6 ^cells, and although this varied slightly with age there was no statistically significant difference between groups. These data indicate that a functional CD8+ T cell response to HCMV consistently develops in early life and is sustained throughout childhood.

### Expression of phenotypic markers (CD28/27 and CD45RA/RO) on HCMV-specific CD8+ cells

To identify the differentiation status of HCMV-specific CD8+ T cells in individual groups, we studied the expression of CD28, CD27, CD45RA, and CD45RO on peripheral blood tetramer+ CD8+ cells using four-color flow cytometry. Both HLA-A2+ and HLA-A24+ subjects were included in this analysis, the results of which are shown in Table 5. These indicate that CD28/CD27 expression on HCMV-specific CD8+ cells was divided amongst 4 subsets. In the youngest three groups (0–4 yr., 5–9 yr., 10–14 yr.), the predominant phenotype of HCMV-specific CD8+ T cells was the CD28-CD27+ T-cell subset. Although the CD28+CD27+ subset, ("early" differentiated), was predominant amongst HCMV specific CD8+ T cells in infants under 12 months (n = 13, data not shown), beyond this time, the CD28-CD27+ subset predominated, and these phenotypes remained relatively stable throughout childhood and early adulthood. However, in the elderly group, the proportion of CD28-CD27- cells ("late differentiated") showed a significant increase. Interestingly, a rare subset of previously unclassified CD28+CD27- T cells population increased with age continuously and represented 22.0% of tetramer+ CD8+ T cells in the elderly people.

**Table 5:** Phenotype (CD27, CD28) of tetramer-positive CD8+ cells in individual groups

Age(yr.) (n = 133)	0–4 (n = 31)	5–9 (n = 24)	10–14 (n = 24)	15–19 (n = 29)	20–29 (n = 13)	60–92 (n = 12)
%						
CD28(-)CD27(+)	43.2 ± 16.8	42.5 ± 14.9	39.1 ± 14.4	31.3 ± 13.7	31.6 ± 11.9	17.2 ± 14.1
CD28(+)CD27(+)	34.9 ± 23.2	28.9 ± 14.5	26.7 ± 13.4	32.8 ± 16.0	40.6 ± 14.9	28.1 ± 17.2
CD28(-)CD27(-)	20.8 ± 17.0	26.0 ± 18.9	30.0 ± 16.9	31.4 ± 20.0	22.3 ± 12.0	40.0 ± 20.5
CD28(+)CD27(-)	1.1 ± 2.5	2.6 ± 3.2	4.2 ± 8.7	4.5 ± 4.7	5.5 ± 6.6	22.0 ± 24.2

Table 6 shows the results of expression of CD45RA and CD45RO on tetramer+ CD8+ T cells. CD45RA+CD45RO- T cells were predominant in all groups. The proportion of each phenotype in individual groups did not change. Therefore, no specific phenotypic patterns of CD45RA and CD45RO related to the age of the subjects were observed in tetramer-positive CD8+ T cells.

**Table 6:** Phenotype (CD45RA, CD45RO) of tetramer-positive CD8+ cells in individual groups

Age(yr.) (n = 131)	0–4 (n = 31)	5–9 (n = 24)	10–14 (n = 24)	15–19 (n = 29)	20–29 (n = 13)	60–92 (n = 12)
%						
CD45RA(-)45RO(+)	12.7 ± 14.5	27.1 ± 22.9	23.3 ± 21.8	27.5 ± 23.5	21.2 ± 19.5	18.0 ± 26.0
CD45RA(+)45RO(+)	30.1 ± 21.8	24.7 ± 16.6	20.0 ± 11.7	21.4 ± 15.9	26.6 ± 18.2	21.2 ± 16.8
CD45RA(-)45RO(-)	12.7 ± 11.0	15.5 ± 12.0	12.1 ± 9.7	15.9 ± 12.7	18.4 ± 15.2	12.2 ± 15.5
CD45RA(+)45RO(-)	43.1 ± 20.5	32.7 ± 14.6	44.5 ± 20.4	35.2 ± 20.6	33.7 ± 18.9	48.5 ± 30.9

## Discussion

In this study, we adapted immunological techniques which have been widely applied to the study of adults to a large pediatric population of over 1000 children. The aim of the study was to define the quantity and quality of antiviral responses in early infection and relate any changes in these to age. Long term longitudinal studies of CMV specific populations are difficult to perform in healthy pediatric populations; nevertheless, using similar large scale cross sectional approaches we have previously observed a clearly dynamic host-virus relationship in adult infection [9,25].

The pattern of age-related accumulation observed in this cross-sectional analysis revealed strong responses in the first year of life, consistent with acquisition of infection during this period. The acquisition of cellular immunity can be regarded as a good marker for viral infection, at a time when serological responses might be confounded by the presence of maternally transmitted antibody.

In particular, we evaluated in detail the frequencies of specific CD8+ T cells during the first four years of life (Table 3, 4). The results showed that initial expansion occurred very early in both HLA-A2 and A24 individuals. These findings support the idea that the primary infection with HCMV commonly occurs during the first or second year of life as suggested in previous studies [2,26-28]. Long-term repetitive antigen stimulation through continuous low level virus reactivation is considered to be responsible for the maintenance of very high levels of CMV specific "effector memory" CD8+ T cells thereafter [6-8]. Why such cells accumulate over time and particularly in the elderly is not known, nor indeed is their efficacy in containing viral reactivation or reinfection. In the pediatric and young adult populations studied here no apparent "inflation" was seen in our analysis, although typical expanded populations were seen in elderly controls. It is possible that "inflation" of responses only occurs after a certain period of host-virus interaction, as is the case in MCMV [8]. Alternatively it may be missed due to the cross-sectional design of the study, Overall however, it does appear that for at least the first couple of decades, the host virus balance remains roughly in status quo.

In this study, responsiveness in both ICS and ELISPOT assays was significantly correlated with tetramer staining. High frequencies of HCMV specific CD8+ cells were observed in even in 0–2 year-old infants, and, although the number of infants examined was relatively small, these findings suggest that infants infected with HCMV have a functional CD8+ T cell response to HCMV similar to that in adults. Moreover, all of these infants but one, who were positive for HCMV tetramer under 1-year-old, were asymptomatic in this study. Those observations are consistent with a recent smaller study which showed that newborns with congenital HCMV infection were asymptomatic and had functional and mature HCMV-specific CD8+ T cells [18,29]. A recent study reported that IFN-γ secretion produced by HCMV-tetramer positive T cells in old individuals was lower than in young individuals [30]. However, the secretion of IFN-γ produced by tetramer reactive cells in elderly individuals was not compared with that in young individuals in this study.

Based on an analysis of CD28/CD27 expression, the stage of "maturation" or "differentiation" of HCMV-specific CD8+ T cells can be defined. CD28+ CD27+ T cells are considered to be naïve cells or early-differentiated T cells, progressing to CD28-CD27- T cells, thought to be fully differentiated T cells [10-14]. We had expected that the predominant phenotype of HCMV-specific CD8+ T cells in the youngest group might be CD28+CD27+. However, the CD28-CD27+, intermediate differentiated phenotype, was predominant (43.2% of CD8+ tetramer positive T cells). Subsets of CD28+CD27+ (naïve or early-differentiated phenotype) and CD28-CD27- (late-differentiated phenotype) represented 34.9% and 20.8% of CD8+ tetramer positive T cells, respectively. The same proportion of HCMV-specific T cells was observed in the 5–9 yr. and 10–14 yr. groups, and also in infants under one-year-old (data not shown). These findings suggest that "mature" CD8+ T cells developed consistently in young infants. In the elderly group, the proportion of CD28-CD27+ (intermediate-differentiated phenotype) T cells was decreased, as the proportion of CD28-CD27- (late-differentiated phenotype) increased substantially. This finding broadly supports the idea that the lineage differentiation pattern of HCMV specific CD8+ cells is CD28+CD27+ → CD28-CD27+→ CD28-CD27-, although it suggests that the movement of cells across this spectrum is not continuous over time. Interestingly, however, we also noted a small proportion of CD28+CD27- T-cells which rose to 22% of tetramer positive CD8+ T-cells in the elderly group.

Naïve CD8+ T cells express CD45RA, and this is uniformly expressed in cord blood. While antigen-experienced CD8+ T cells initially express CD45RO, re-expression of CD45RA may occur and this has been described as a state of late or terminal differentiation – although longitudinal studies of single cells have not been performed. According to this scheme, it was expected that a subset of CD45RA+CD45RO- tetramer+ cells would accumulate with age. In contrast, the CD45RA+CD45RO- subset was predominant in all of the age-groups, even in the youngest children and the proportion did not change through life. There are two possibilities to explain these observations. First, this state may be driven by continuous interaction with antigen. However, this hypothesis is not entirely consistent with the results of CD28/CD27 subsets of this study, in which CD28-CD27- "late" differentiated cells, were predominant in only the elderly. Alternatively, the levels of CD45 isoforms expression correlate poorly with the stages of antigen-driven T cell differentiation. This is supported by a previous study, which showed that CD45RA expression was not correlated to the differentiation phenotypes (28). Expression of other cell surface markers, such as CCR7, CD57 or CD85j might correlate more closely with the evolution of T cell responses over time [31-37].

In this study, HLA-A2+ subjects were selected by flow cytometry using the HLA-A2 mAb. However, the HLA-A2 molecular type of Japanese is classified into three subtypes. HLA-A*0201, A*0206, and A*0207 representing 10%, 10%, and 3%, of Japanese, respectively [23]. Because the difference among HLA subtypes may in principle affect the antigen processing and the presentation of CTL epitopes in the context of HLA class I molecules, the magnitude of CTL response might depend on HLA subtype [38]. However, the frequency of individuals staining positive for the HLA-A2 pp65 tetramer was higher than that in a previous study, in a Caucasian population expressing predominantly HLA-A*0201 [9]. In addition, there was no difference in the percentage of tetramer/CD8 in the 20–29-year-old group between the previous study and this study. Those observations suggest that the difference among the three subtypes, did not substantially influence the results of tetramer staining. However, further studies are required to determine whether the difference among HLA-A2 subtypes can influence functional assays.

CMV sero-status of subjects was not evaluated in this study. We reported that 77% of HCMV-seropositive HLA-A2 subjects were positive for CMV tetramer [9]. These findings suggest that the result of tetramer assay can be related to sero-status, because tetramer reactive T cells are memory T cells. However, further studies are required to assess whether the results of tetramer assays are consistent with the HCMV sero-status in different population.

In previous study [9], we showed "memory inflation" in an HLA-A2 positive population. However, the subject was a Caucasioan population. Therefore, there was a possibility that the frequency of HCMV specific tetramer might be different in a Japanese population. In order to confirm "memory inflation" in Japanese, HLA-A2 positive elderly subjects were tested. In this study, we could confirm that "memory inflation" was present in a Japanese population. However, HLA-A24 elderly subjects were not tested because HLA-A24 subjects were not examined in our previous study.

In conclusion, the maintenance of large populations of HCMV-specific CD8+ T cells was observed throughout childhood, with substantial responses to infection in early infancy. Overall major age-related changes in tetramer+ CD8+ T cells were not observed in the first two decades of life and tetramer+ CD8+ cells of young infants showed a mature phenotype and function similar to that in adults. These data provide novel insight into the relative maturity of the infant antiviral response, and the impact of CMV on the childhood immune system. Additionally, they demonstrate the technical feasibility of detailed cellular immunological studies in pediatric populations, undertaken at a large scale as may be applicable in other childhood infections and vaccine programs.

## Abbreviations

HCMV, human cytomegalovirus

MCMV, murine cytomegalovirus

Tetramer, tetrameric complexes

ELISPOT, enzyme-linked immunospot

ICS, intracellular cytokine staining

HLA, human leukocytic antigen

FITC, fluorescein isothiocyanate

PerCP, peridinin chlorophyll protein

APC, allophycocyanin

PE, phycoerythrin

PBMCs, peripheral mononuclear cells

## Authors' contributions

HK, SS, JN, ML, AV and PK conceived and designed the experiments. HK performed the experiments. HK, AI, TS, TF, SN, and HN participated in data collection. HK and JN analysed the data. HK and PK wrote this paper.

## Conflicts of interest

The author(s) declare that they have no competing interests.
